# Human Papillomavirus Types Associated with Cervical Dysplasia among HIV- and Non-HIV-Infected Women Attending Reproductive Health Clinics in Eastern Kenya

**DOI:** 10.1155/2021/2250690

**Published:** 2021-09-20

**Authors:** James Kinoti Njue, Margaret Muturi, Lucy Kamau, Raphael Lwembe

**Affiliations:** ^1^Department of Medical Laboratory Sciences, Kenyatta University, Kenya; ^2^Centre for Virus Research, Kenya Medical Research Institute (KEMRI), Kenya

## Abstract

**Background:**

Human papillomavirus (HPV) causes over 99% of all cervical cancer globally. In 2019, it was responsible for 3286 deaths in Kenya. Data on the epidemiological distribution of HPV genotypes by cervical dysplasia and HIV-infected women which is important in designing prevention strategy monitoring treatment and management of cervical cancer is lacking in Eastern Kenya.

**Objective:**

To determine HPV genotype prevalence and their association with cervical dysplasia among HIV-infected (cases) and noninfected (control) women aged 18-48 years seeking reproductive healthcare.

**Methods:**

A cervical broom was softly rotated 360 degrees five times to exfoliate cells from the region of the transformation zone, squamocolumnar junction, and endocervical canal for HPV genotyping. Social-demographic and risk factors responsible for HPV acquisition were collected using a questionnaire. Laboratory outcome and questionnaire data statistical relationships were computed using Pearson chi-square test.

**Results:**

317 women (cases: 161 (50.8%), control 156 (49.2%), mean age: 34.3,SD ± 10.4, range 18-46 years) were recruited from Embu (85/317 (26.8%)), Isiolo (64/317 (20.2%)), Kirinyaga (56/317 (17.7%)), Meru (81/317 (25.6%)), and Tharaka-Nithi (31/317 (9.8%)). The frequency HPV genotypes detected by cervical dysplasia were CIN1 (cases: HPV81 (12/317 (3.8%)), HPV11 (2/317 (0.6%)); control: HPV53 and 66 coinfection (1/317 (0.3%)), CIN2 (cases: HPV11, HPV16, HPV66 ((1/317 (0.3%) each), HPV81 (6/317 (1.9%)), and single case (1/317 (0.3%)) of HPV11 and 66, HPV81 and 44, HPV81 and 88, HPV9 and 53, and HPV16 and 58 coinfection; control: HPV81 (2/317 (0.6%)) and invasive cervical cancer (cases: HPV16 (1/317 (0.3%)) and HPV81 (3/317 (0.9%)); control: HPV16 and 66 (1/317 (0.3%))).

**Conclusions:**

There was a higher frequency of both high-risk and low-risk HPV genotypes associated with cervical dysplasia among HIV-infected than HIV-uninfected women seeking reproductive health care. This study provides epidemiological data on the existence of nonvaccine HPV types associated with cervical dysplasia in the region.

## 1. Introduction

Human papillomavirus (HPV) is primarily responsible for 99.7% of cervical cancers globally [1]. It can be sexually transmitted and causes cervical neoplastic changes leading to cervical cancer and is the second type of cancer in women aged 15-44 years in Kenya [1, 2]. It was responsible for 311,365 (8.2%) annual global mortality, 37,017 (16.9%) in East Africa, and 3250 (12.8%) in Kenya in 2019 [1, 2]. The cervical screening rate in Kenya is only 3.2% for women aged over 18 years [2].

Human papillomaviruses are grouped based on their oncogenicity as Group I: “Carcinogenic” and include HPV16, 18, 31, 33, 35, 39, 45, 51, 52, 56, 58, and 59; Group 2A: “probably-carcinogenic” which includes HPV26, 53, 66, 67, 70, 73, 82, 30, 34, 69, 85 and 97; Group 2B: “possibly-carcinogenic” which includes HPV6 and 11; and Group 3: “unclassifiable to carcinogenicity” [2, 3].

Oncogenicity of HPV is further increased by immunocompromised status following human immunodeficiency virus (HIV) infection, long-term exposure to hormonal-contraceptives, and autoimmune drugs which favor tumorigenic effects of HPV-genome and weaken the immune system responsible of clearing cancer cells [3, 4]. Other risk factors include early sex debut, long-term inflammation caused by recurrent genital infections, hormonal changes during pregnancy, cervical trauma during labor, and failure to undergo HPV vaccination [3–5].

Literatures review wide spectra of HPV genotypes associated with cervical dysplasia among HIV-infected women [1, 5–7]. No such data is available from Eastern Kenya which has implications regarding cervical cancer burden and efficacy of HPV vaccination in the region [6]. Cross-protection between HPV types in the current vaccine and those that are predominant in the population has also not been established. This study therefore aimed at determining HPV types associated with cervical dysplasia among HIV-infected and noninfected women attending reproductive health clinics in the region.

## 2. Methods

### 2.1. Study Design and Participants

This cross-sectional study involved 317 women aged 18-46 years who attended reproductive health clinics in referral hospitals of Isiolo, Kirinyaga, Meru, Tharaka-Nithi, and Embu Counties in Eastern Kenya from January to December 2019. A sample size was calculated by using an ICO HPV infection rate (1) of 2.8% (total: 158 cases and 158 controls). Samples were distributed according to reproductive health clinic's diagnosis 27 VIA/VILLI-positive cases starting July–December 2017 in the region. The stratified technique of sampling was used to obtain five strata based on county of residence then simple random sampling was then used to recruit the required number of a participant in each stratum.

### 2.2. Inclusion and Exclusion Criteria

Inclusion criteria are as follows: consenting women aged 18-46 years from Eastern Kenya.

Exclusion criteria are as follows: menstruating and pregnant women, mentally incompetent, and those with eroded cervix or history of ablative procedures or medical treatment for cervical disease in the last six months [6, 7].

### 2.3. Detection of Cervical Dysplasia

A two-step approach was applied where visual inspection with acetic acid solution (VIA) was performed followed by Lugol's iodine solution (VILLI) which are based on the colors taken up by the cervical transformation zone [8, 9]. The outcome was reported as VIA/VILLI positive or negative.

### 2.4. Social-Demographic Data Collection

Social-demographic data was collected from participants with precaution after consenting using a questionnaire. The questionnaire focused on data on the residence, age, education level, parity, sexual orientation, and choice of family planning method.

### 2.5. HIV Determination

HIV serostatus was determined as per national algorithm. Alere Determine®HIV-1/2 test by Abbort Co. was used as baseline screening while the First Response® HIV 1-2-0 card test by Premier Medical Corporation was used as a confirmatory test in case of discrepancies. *Uni-Gold™* Recombigen® *HIV-1/2 by* Trinity Biotech was used as a tie breaker test [10].

### 2.6. Collection and Storage of Cervical Exfoliated Cell Samples

External genitalia were examined while the participant lays in a lithotomic position. A speculum was rinsed in warm water, lubricated, and used to locate the cervical opening (os) under direct light. The mucus plug in os was removed and wiped to ensure sufficient cells were collected. A cervical broom (Dacron cervical broom; Digene Corporation, Silver Spring, Maryland STM™) was softly rotated 360 degrees five times to exfoliate cells from the region of the transformation zone, squamocolumnar junction, and endocervical canal [9]. Exfoliated cells were spread evenly and fixed immediately on a clean glass slide. The broom bristles were then dipped into aqueous minimum essential media (MEM) and the broom handle snapped so that it remained in the tightly closed vial and stored at 1-4°C [2, 5, 8].

### 2.7. Cytology

A standardized protocol for cytopathology request form [5, 6], Pap smears staining, and examination [6, 9] was followed for detection of nuclei and cytoplasm cytological changes following HPV infection. Cytopathologists supervised by a pathologist at Embu and Meru Hospitals were required to fill a pathology synoptic reporting form [6] using the Bethesda 2001 guidelines for reporting slides using a binocular microscope. Slides were later transferred to Kenya Medical Research Institute (KEMRI) for examination by pathologist to ensure the quality of cytology results. Pap smear results were classified as normal or abnormal (ASCUS, CIN1, CIN2, CIN3, or ICC). Cervical infections (candidiasis, cervicitis, trachomatis, and bacterial vaginitis) reported in cytology were exempted from the study [9, 11, 12].

### 2.8. HPV DNA Extraction and Amplification

All samples stored for HPV DNA detection were analyzed by the following procedure:

- DNA extraction

A 96-well format HighPrep™ Viral-DNA/RNA, MagBio Genomics, Inc. USA/Canada Lysis kit was used. Master Mix was prepared by mixing 240 *μ*L Lysis-buffer, 8 *μ*LRNA carrier, and 280 *μ*L isopropanol sample/volume. Samples stored at 1-4°C were thawed, vortexed for 5 minutes, and then centrifuged at 10000 r/min for 5 minutes to extract cytological material from the brush into MEM. Samples were subjected to the kit protocol to obtain DNA extracts by magnetic bead technique The eluate was stored at -20°C [13, 14].

(b) HPV DNA PCR

HPV detection was achieved by amplifying an L1 portion of the HPV genome that is relatively conserved through L1 consensus nested PCR in the ABI-thermocycler Model 9600; Applied Biosystems®. HPV consensus primary primers PGMY09 (GCACAGGGACATAACAATGG) and PGMY11 (CGTCCCAAAGGAAACTGATC) [15] that target the 450 bp region in the L1 ORF; the genome was included in the first PCR reaction. Additional primer sets targeting the same region of L1, MGP5+(ACGTTGGATGTTTGTTACTGTGGTGGATACTAC) and MGP6+(ACGTTGGATGGAAAAATAAACTGTAAATCATATTCCT) were used to produce shorter amplicon of ~160 bp [15]. 5 *μ*M working stock of each primer was prepared by adding 50 *μ*L biotinylated PGMY09 100 *μ*M to 350 *μ*L nanopure water and 50 *μ*L PGMY11 100 *μ*M primers to 750 *μ*L nanopure water. They were later distributed each 5 *μ*M working stock in 45–90 *μ*L aliquots and stored at -20°C. In the primary PCR, 5 *μ*L of the extract was amplified in a Master Mix containing PCR buffer (1x), 2.0 mM MgCl_2_, 100 *μ*M dNTPs, 0.13 parts *Taq* polymerase enzyme and 500 nM MY09, and 500 nM MY11 (forward and reverse primer, respectively). First reaction: 4 minutes at 95°C (initial denaturation) then 30 cycles of 20 seconds at 95°C, 40 seconds at 56°C, and 2 minutes at 72°C, and final extension for 7 minutes at 72°C. In nested PCR, 5 *μ*L of the first PCR product, 2 mM MgCl_2_, 500 nM GP5+, and 500 nM GP6+ (forward and reverse primer, respectively), 400 *μ*M of dNTPs, and 0.13 units of *Taq* polymerase enzyme composed the Master Mix. Nested reaction: 4 minutes at 95°C (initial denaturation) then 30 cycles of 20 seconds at 95°C, 40 seconds at 60°C, 40 minutes at 72°C, and final extension for 7 minutes at 72°C. Positive control of CIN2+ and negative control of distilled water were incorporated in all primer cycles [15].

(c) Gel electrophoresis and UV visualization

Tris-Borate-EDTA 10x was prepared by dissolving 162 g Tris-base, 50 g boric acid, and 9.5 g EDTA in nanopure water to 1.0 liter volume (pH 8.8). 5 *μ*L PCR-product in 4% agarose was used in gel electrophoresis where samples that showed a band of 160 bp were considered positive. The positive PCR product was purified using the QIAquick DNA purification kit™ (Qiagen, Germany) before sequencing [7, 13].

(d) HPV DNA sequencing

DNA sequencing was performed in ABI-thermocycler Model 9600 (Applied Biosystems) for 20 reaction cycles of 1 *μ*L positive PCR-product, 1 *μ*L of 5 *μ*M GP6+ primer, 1 *μ*L BigDye® Terminator, 3.5 *μ*L buffer (5x), and 13.5 mL nanopure water according to the protocol supplied by the manufacturer. Centri-Sep column (Princeton Separations, Adelphia, NJ) was used for dye-terminator cleanup; then, the reaction mixture was sequenced in an automated ABI 3130 four-capillary genetic analyzer [15, 16].

(e) HPV genotyping and phylogenetic analysis

CHROMAS software Version 2.4.3 was used to edit the sequences obtained. HPV sequences were blasted in NCBI http://blast.ncbi.nlm.gov/blast.cgi. Sequences with unique divergences from the same HPV type underwent phylogenic analysis, and references were obtained from GenBank. The input file of representative sequences and their references was made and subjected for multiple alignments with CLUSTAL W in MEGA X software [15, 16]. The maximum likelihood method and the Tamura-Nei model were used to infer evolutionary history. Neighbor-Join and BioNJ algorithms were used to construct initial trees for the heuristic search of the matrix of pairwise distances by the maximum composite likelihood (MCL) method by selecting the topology with superior log likelihood value. Eighty-six nucleotide sequences were involved while codon positions were 1st+2nd+3^rd^ [16–18].

### 2.9. Ethics Approval

This study was approved by the KEMRI Scientific Ethical Review Unit (approval number: KEMRI/SERU/CVR/004/3342). Participants were consented orally where all steps and procedures for HIV and cervical exfoliated cell sample collection; analysis and collection of their results were explained. Laboratory and questionnaire data were confidentially stored by the principal investigator.

## 3. Results

### 3.1. Distribution of HPV Genotypes among HIV- and Non-HIV-Infected Women (*N* = 106)

**Figure 1:**
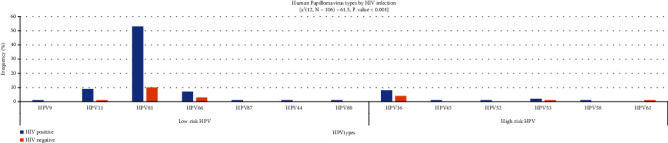
Distribution of HPV genotypes among HIV- and non-HIV-infected women (*n* = 106).

Thirteen HPV types detected were low-risk HPV9, HPV11, HPV81, HPV66, HPV87, and HPV88 while high-risk types were HPV16, HPV53, HPV61, HPV45, HPV52, and HPV58. There was significant association between HPV types detected and HIV serostatus (*p* < 0.001, *df* = 12) (Figure 1).

### 3.2. Distribution of HPV Genotypes with Cytology (Levels of Cervical Dysplasia) among HIV- and Non-HIV-Infected Women

A total of 62 (19.2%) HIV-infected women were infected with single HPV type compared to (93.2%) HIV-uninfected women while 73 (23.03%) were infected by multiple HPV types compared with 13 (4.1%) HIV-uninfected women (*p* < .001, *df* = 1) (Table 1).

### 3.3. Social Demographic and HPV-Associated Risk Factors among HIV- and Non-HIV-Infected Women

A total of 317 (mean age: 34.3years, *SD* ± 10.4, range 18-46) women were recruited. Overall HPV prevalence was 27.1% ((86/317); cases: 23.2% (73/317) and control: 4.1% (13/317)). There was significant association between HPV infection and age, parity, education level, and reported number of sex partners (Table 2).

### 3.4. Distribution of HPV Genotypes among HIV- and Non-HIV-Infected Women

HPV type distribution among HIV-infected women was significantly associated by residence (Embu (*p* = 0.042), Isiolo (*p* < 0.001), Meru (*p* < 0.001), and Tharaka-Nithi (*p* = 0.048)), age (≤30 years (*p* < 0.001); <30 years (*p* = 0.017)), parity (≤35 (*p* = 0.031); >35 (*p* < 0.001)), family planning method (hormonal (*p* = 0.047, *df* = 1); nonhormonal contraceptives (*p* < 0.001) use), and number of sex partners (1 (*p* < 0.001; >1 (*p* < 0.001)) (Table 3).

### 3.5. Association of Cervical Cytology with Other Clinical Reproductive Health Ailments

**Figure 2:**
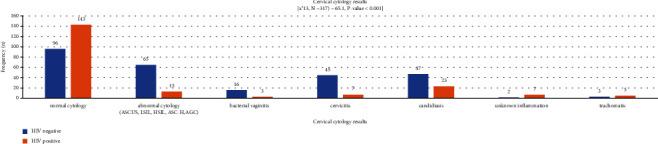
Associated of cervical cytology with other clinical reproductive health ailments.

A total of 96 (30.3%) HIV-positive women had normal cytology as compared to 143 (45.1%) HIV-negative, whereas 65 (20.5%) HIV-positive women had abnormal cytology results compared with 13 (4.1%) HIV-negative (*p* = 0.001) (Figure 2).

### 3.6. Association of HPV Genotypes with Cervical Dysplasia among HIV-Infected Women and Noninfected Women

Frequency of common HPV genotypes detected by cervical dysplasia among HIV-infected women were as follows: CIN1 (17 (5.4%)): HPV81 (12 (3.8%)), HPV11 (2 (0.6%)), CIN2 (16 (5.0%)): single case (0.3%) of HPV11, HPV16, and HPV66 and HPV81 (6 (1.9%)), CIN3 (6 (1.8%)): HPV11 (1 (0.3%)), HPV81 (4 (1.2%)) and invasive cancer (5 (1.5%)): HPV16 (1 (0.3%)) and HPV81 (3 (0.9%)), (*p* < 0.001) (Table 4).

### 3.7. Distribution of HPV Genotypes among HIV- and Non-HIV-Infected Women with Cervical Dysplasia

**Figure 3:**
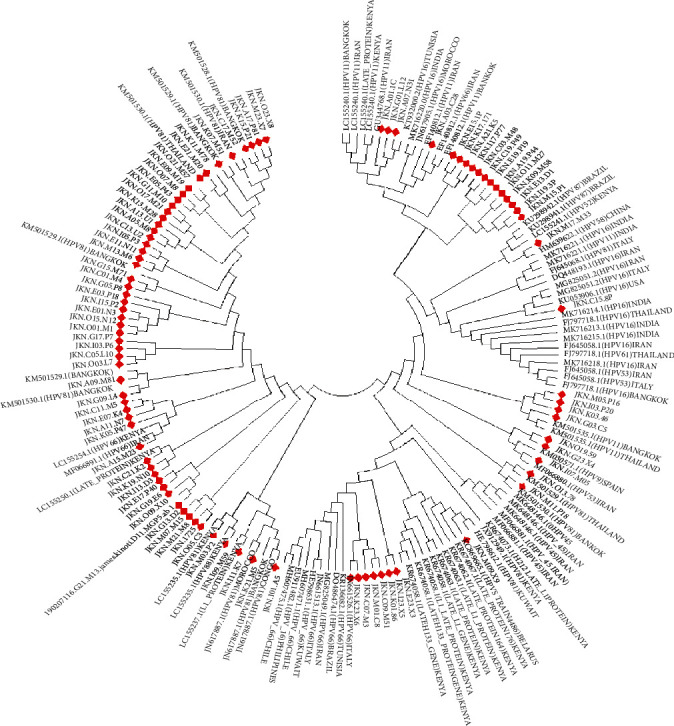
Distribution of HPV genotypes among HIV- and non-HIV-infected women with cervical dysplasia.

Phylogenetic tree of HPV samples marked in red aligned against the representation of the different HPV genotypes and subtypes distributed in different regions worldwide, constructed using MEGA6 neighbor-joining using 1000 bootstrap reference method. Most HPV81 clustered with those cases was detected in Bangkok Morocco and Thailand, HPV66 clustered with those from Kenya and Iran, while HPV66 clustered with cases reported in Tunisia, Morocco, Iran, and India (Figure 3).

## 4. Discussion

This study established an overall HPV infection rate of 27.12% (cases: 73 (23.03%); control: 13 (4.1%)). It agrees with the overall age-specific HPV infection rate of 27% in neighbouring Nairobi region [1]. HIV infection was significantly associated with HPV infection. A high HPV prevalence was reported in Embu and Meru Counties where HIV was most prevalent. There is a need to establish HPV prevalence in other regions of the country with high HIV burden [19] that could be harbouring more HPV infection rate, hence raising overall national HPV prevalence (40%) [1]. This indicator should be prioritised by public health interventions to reduce cervical cancer morbidity and mortality.

Women aged below 35 years of age had a high rate of mixed HPV genotypes and a significant association between HIV infection and abnormal cytology outcome which agrees with published observations [5]. A possible explanation is that HIV infection may have facilitated cervical dysplasia in young women, and an inverse relationship of high-risk HPV prevalence and age has been described [20].

A high infection rate was also noted among Christians who comprised the majority of participants than Muslims. This study concurs with other studies that have shown high HPV infection rate among secondary school educated women. This is attributed to a higher risky sexual behaviour and low knowledge on HPV and associated risk factors as reported among young women in South Africa [20, 21].

HPV infection rate was high among HIV-infected women with high parity, married, and those using hormonal contraceptive than other categories. This could be associated with high persistence and subsequent low clearance of HPV upon infection among these groups as reported by other studies [5, 22].

There was a significant association between having multiple sex partners and HPV infection. Most of the participants in this category were HPV infected. This could be because having multiple sex partners increased their chance of contracting HPV and HIV. The dominance of HPV infection in normal cytology was detailed high (28.8%) as published [1]. Younger women showed a higher infection rate in normal cytology than their older counterparts. This is because they are more sexually active and highly likely to encounter wider spectra of HPV types [7, 15]. However, they are reported to have a high HPV clearance rate than their older counterparts [23] hence less likely to develop cervical dysplasia.

The foremost common HPV detected in the region was lrHPV81—*Alphapapillomavirus* 3 (*α*3) HPV species by both HIV serostatus and cytology outcomes. The predominance of HPV81 (CP8304) has moreover been detailed in women with anomalous cytology in Kenya [6, 7] and Qatar [18]. It is associated with precancerous and cancerous lesions and mostly detected among immunocompromised women [7, 24, 25] as seen in this study.

HPV16 (*α*9) and HPV66 (*α*6) were most common high-risk types associated with invasive cancer by HIV serostatus. Their predominance in abnormal cytology among HIV-infected women has been established [7, 15, 24, 25]. HPV66 is classified as oncogenic because of its close phylogenetic relationship with HPV56 (*α*9). This study and others therefore recommend routine testing for HPV16, HPV52, and HPV66 [20] since these members of (*α*7) and (*α*9) dominate neoplastic tissues. Though they were also detected in normal cytology, a study has demonstrated the genetic variability and frequent mutating L1, E6, and E7 genes mostly HPV52 and HPV53 (*α*7) resulting into intratypic variants with increased oncogenic potential [21].

Single HPV type infection in CIN1+ showed diversity compared with multiple HPV type's infection in CIN1+ by HIV infection. Diverse infection was also seen among HIV-infected women with multiple HPV infections. Other common lrHPV types detected were HPV11, HPV44 (*α*10), and HPV88 which do not feature in many studies as potential oncogenic types Here, their association with CIN+ could have been increased by HIV infection as reported in other studies [5, 15, 21].

There were a significantly low number of participants infected with HPV types of the same alpha group in this study. There were also a reduced number of hrHPV52 and 58 and absence of most of other hrHPV types, notably HPV18. This could be attributed to antibodies' cross-reaction which is achieved within species unlike across species. Antibodies against HPV16 cross-react and protect against HPV52 and HPV58 of the same *α*9 species, HPV18 cross-react with HPV66, and HPV53 (*α*7) species and HPV81 will cross-react with HPV61 [26].

This study and literature data in Kenya establish the predominance of mixed HPV types other than those included in the current vaccine [15]. This puts doubt on the effectiveness of the current vaccine in protection and/or cross-protection against hrHPV among women with compromised immunity.

The phylogenetic trees illustrated that single or multiple HPV types infected each participant as shown by 15 participants. Another reason is that HPV genes are replicated by the host replication machinery suggesting that a very low human autosomal–like mutation rate would be operating as established in another study [21].

## 5. Limitation

The study group is a minimal presentation, and data presented cannot be generalized as exact outcome if all women of reproductive age in Eastern Kenya were sampled. Responses to the questionnaire could have been altered by missing key words like ‘cervical cancer' in local direct during translation but caution was taken to give accurate translation and meaning.

## 6. Conclusions

There was a higher frequency of both high-risk and low-risk HPV genotypes associated with cervical dysplasia among HIV-infected than HIV-uninfected women seeking reproductive health care. This study provides epidemiological data on the existence of nonvaccine HPV types associated with cervical dysplasia in Eastern Kenya. Genotypic HPV diversity established has potential impact on new vaccines design and need for increased cervical screening among HIV-infected women.

## Figures and Tables

**Table 1:** Total HPV genotypes prevalence identified as single or multiple infections.

Infection type	*N* (%)	HIV negative	HIV positive	*p* value
Single HPV type infection				
Low-risk type	67 (21.1)	9 (2.8): HPV81	7 (2.2): HPV11	<0.001^∗∗^
			51 (16.1): HPV81	
High-risk type	4 (1.3)		3 (0.1): HPV16	
			1 (0.3): HPV66	
Total (single HPV types)	71 (22.4)	9 (3.2)	62 (19.2)	
Multiple HPV types of infection				
Low-risk type	2 (0.6)		1 (0.3): HPV81, 44	
			1 (0.3): HPV81, 88	

High-risk type	9 (2.8)	3 (0.9): HPV16, 66	4 (1.3): HPV16, 66	
			1 (0.3): HPV53, 66	
			1 (0.3): HPV16, 58	

Low- and high-risk type	4 (1.2)	1 (0.3): HPV11, 16, 53, 61, 81	1 (0.3): HPV9, 53	
			1 (0.3): HPV11, 66	
			1 (0.3): HPV11, 45, 52, 87	
Total (multiple HPV types)	15 (4.7)	4 (1.2)	11 (3.5)	
Total (*N* = 317)	86 (27.12)	13 (4.1)	73 (23.03)	

^∗∗^: the probability at the 0.001 level.

**Table 2:** Sociodemographic and HPV-associated risk factors among HIV- and non-HIV-infected women.

Category	*N* (%)	Total	HIV negative (*n* (%))				HIV positive (*n* (%)]			
		HPV	HPV status		Total	*p* value	HPV status		Total	*p* value
		Prevalence	Negative	positive			negative	positive		
Residence										
Embu	85 (26.8)	27 (8.5)	36 (11.4)	5 (1.6)	41 (13.0)	0.853	22 (6.9)	22 (6.9)	44 (13.8)	0.383
Isiolo	64 (20.2)	18 (5.7)	35 (11.0)	3 (0.9)	38 (11.9)		11 (3.5)	15 (4.7)	26 (8.2)	
Kirinyaga	56 (17.7)	12 (3.8)	22 (6.9)	1 (0.3)	23 (7.2)		22 (6.9)	11 (3.5)	33 (10.4)	
Meru	81 (25.6)	20 (6.3)	37 (11.7)	3 (0.9)	40 (12.6)		24 (7.6)	17 (5.4)	41 (13.0)	
T.Nithi	31 (9.8)	9 (2.8)	13 (4.1)	1 (0.3)	14 (4.4)		9 (2.8)	5)	17 (5.3)	
Age (mean: 34.3, range 18-46)										
<35	162 (51.2)	60 (18.9)	59 (18.6)	10 (3.2)	69 (21.8)	0.014^∗^	43 (13.6)	50 (15.8)	93 (29.4)	0.009^∗^
>35	155 (48.8)	26 (8.2)	84 (26.5)	3 (0.9)	87 (27.4)		45 (14.2)	23 (7.3)	68 (21.5)	
Religion										
Christian	255 (80.4)	70 (22.1)	108 (34.1)	10 (3.1)	118 (37)	0.607	77 (24.3)	60 (18.9)	137 (43.2)	0.237
Muslim	62 (19.6)	16 (5.0)	35 (11.1)	3 (0.9)	38 (12.0)		11 (3.5)	13 (4.1)	24 (7.6)	
Education level										
Primary	96 (30.3)	20 (6.3)	40 (12.6)	3 (0.9)	43 (13.5)	0.115	36 (11.4)	17 (5.4)	53 (16.8)	0.004^∗^
Secondary	135 (42.6)	39 (12.3)	66 (20.8)	4 (1.3)	70 (22.1)		30 (9.5)	35 (11.0)	65 (19.5)	
College	67 (21.1)	21 (6.6)	33 (10.4)	4 (1.3)	37 (11.7)		13 (4.1)	17 (5.4)	30 (9.5)	
University	19 (6.0)	6 (1.9)	4 (1.3)	2 (0.6)	6 (1.9)		9 (2.8)	4 (1.3)	13 (4.2)	
Contraceptive use										
Other	223 (70.3)	66 (20.8)	95 (30.0)	8 (2.5)	103 (32.5)	0.469	62 (19.6)	58 (18.3)	120 (37.9)	0.131
Hormonal	94 (29.7)	20 (6.3)	48 (15.1)	5 (1.6)	53 (16.7)		26 (8.2)	15 (4.7)	41 (12.7)	
Parity										
>3	68 (21.5)	16 (5.0)	30 (9.5)	8 (2.5)	38 (12.0)	0.003^∗^	22 (6.9)	39 (12.3)	41 (39.2)	0.001^∗∗^
<3	249 (78.5)	70 (22.1)	113 (35.6)	5 (1.6)	118 (37.2)		66 (20.8)	34 (10.7)	100 (21.5)	
Marital status										
Married	226 (71.3)	55 (17.4)	109 (34.4)	8 (2.5)	117 (36.9)	0.249	62 (19.6)	47 (14.8)	109 (34.4)	0.299
Separated	32 (10.1)	8 (2.5)	13 (4.1)	1 (0.3)	14 (4.4)		11 (3.5)	7 (2.2)	18 (5.7)	
Single	41 (12.9)	12 (3.8)	17 (5.4)	2 (0.6)	19 (6.0)		12 (3.8)	10 (3.2)	22 (7.0)	
Divorced	6 (1.9)	3 (0.9)	2 (0.6)	1 (0.3)	3 (0.9)		1 (0.3)	2 (0.6)	3 (0.9)	
Widowed	12 (3.8)	8 (2.5)	2 (0.6)	1 (0.3)	3 (0.9)		2 (0.6)	7 (2.2)	9 (2.8)	
Sex partners										
1	186 (58.7)	43 (13.6)	91 (28.7)	7 (2.2)	98 (30.9)	0.339	52 (16.4)	36 (11.4)	88 (27.8)	0.014^∗^
>1	131 (41.3)	43 (13.6)	52 (16.4)	6 (1.9)	58 (18.3)		36 (11.4)	37 (11.7)	73 (23.1)	
										
Total	317 (100.0)	86 (27.1)	143 (45.1)	13 (4.1)	156 (49.2)		88 (27.8)	73 (23.2)	161 (50.8)	0.001^∗^

N: negative; P: positive; ^∗∗^: the probability at the 0.001 level; ^∗^: the probability at the 0.005 level.

**Table 3:** Distribution of HPV genotypes among HIV- and non-HIV-infected women.

Category	HIV test	HPV infection													*t*otal	*p* value
		High-risk HPV types					Low-risk HPV types									
		16	45	53	58	66	9	11	44	52	61	81	87	88		
Residence																
Embu	N	1 (0.9)				1 (0.9)						4 (3.8)			6 (5.7)	0.042^∗^
	P	3 (2.8)	1 (0.9)	2 (1.9)	1 (0.9)	2 (1.9)	1 (0.9)	4 (3.8)		1 (0.9)		13 (12.8)	1 (0.9)		29 (27.6)		
Isiolo	N	2 (1.9)				3 (2.8)						1 (0.9)			6 (5.7)	0.001^∗∗^	
	P	1 (0.9)				3 (2.8)		3 (2.8)	1 (0.9)			11 (10.5)		1 (0.9)	20 (19.0)		
Kirinyaga	N											1 (0.9)			1 (0.9)	0.076	
	P	2 (1.9)										8 (7.6)			10 (9.5)		
Meru	N	1 (0.9)		1 (0.9)				1 (0.9)			1 (0.9)	3 (2.8)			7 (6.6)	0.001^∗∗^	
	P	2 (1.9)										15 (14.3)			17 (16.2)		
T.Nithi	N											1 (0.9)			1 (0.9)	0.048^∗^	
	P							2 (1.9)				6 (5.7)			8 (7.6)		
Age																	
<35	N	3 (2.8)		1 (0.9)		2 (1.9)		1 (0.9)				5 (4.8)			12 (11.4)	0.001∗∗	
	P	3 (2.8)	1 (0.9)	2 (1.9)		5 (4.8)	1 (0.9)	8 (7.6)		1 (0.9)		39 (37.1)	1 (0.9)		61 (58.1)		
>35	N	1 (0.9)			1 (0.9)	1 (0.9)						4 (3.8)			7 (6.6)	0.017∗∗	
	P	5 (4.8)				2 (1.9)		1 (0.9)	1 (0.9)		1 (0.9)	14 (13.3)			24 (22.8)		
Family planning																	
hormonal	N	1 (0.9)				1 (0.9)						4 (3.8)			6 (5.7)	0.047^∗^	
	P	1 (0.9)		2 (1.9)		3 (2.8)	1 (0.9)	2 (1.9)				10 (9.5)			19 (18.1)		
Other	N	3 (2.8)		1 (0.9)		2 (1.9)		1 (0.9)			1 (0.9)	5 (4.8)			13 (12.8)	0.001^∗∗^	
	P	7 (6.6)	1 (0.9)		1 (0.9)	4 (3.8)		7 (6.6)	1 (0.9)	1 (0.9)		43 (40.9)	1 (0.9)	1 (0.9)	67 (63.8)		
Parity																	
<3	N	1 (0.9)				1 (0.9)						2 (1.9)			4 (3.8)	0.001^∗∗^	
	P	2 (1.9)				1 (0.9)		3 (2.8)	1 (0.9)			8 (7.6)			15 (14.3)		
>3	N	3 (2.8)		1 (0.9)		2 (1.9)		1 (0.9)				7 (6.6)			14 (13.3)		
	P	6 (5.7)	1 (0.9)	2 (1.9)	1 (0.9)	6 (5.7)	1 (0.9)	6 (5.7)		1 (0.9)	1 (0.9)	45 (42.8)	1 (0.9)	1 (0.9)	72 (68.6)		
Number of sex partners																	
One	N	3 (2.8)				3 (2.8)						4 (3.8)			10 (9.5)	0.001^∗∗^	
	P	3 (2.8)	1 (0.9)		1 (0.9)	1 (0.9)		5 (4.8)		1 (0.9)		28 (26.8)	1 (0.9)	1 (0.9)	42 (40.0)		
>one	N	1 (0.9)						1 (0.9)			1 (0.9)	5 (4.8)			8 (7.6)	0.001^∗∗^	
	P	5 (4.8)		1 (0.9)		6 (5.7)	1 (0.9)	4 (3.8)	1 (0.9)			25 (23.8)			43 (40.9)		
Total	N	4 (3.8)				3 (2.8)		1 (0.9)			1 (0.9)	9 (8.6)			18 (17.1)	0.001	
	P	8 (7.6)	1 (0.9)	1 (0.9)	1 (0.9)	7 (6.6)	1 (0.9)	9 (8.6)	1 (0.9)	1 (0.9)		53 (50.5)	1 (0.9)	1 (0.9)	85 (80.9)		

N: negative; P: positive; ^∗∗^: the probability at the 0.001 level; ^∗^: the probability at the 0.005 level; T. Nithi: Tharaka-Nithi County.

**Table 4:** HIV types detected by Pap smear results.

HIV	HPV type of infection	Normal cytology (*n* (%))	Abnormal cytology (*n* (%))					Total	*p* value
			ASCUS	CIN 1	CIN2	CIN3	ICC		
Negative	Single type								
	HPV81	3 (0.9)	1 (0.3)	2 (0.6)	2 (0.6)	1 (0.3)		9 (2.7)	0.001^∗^
	Multiple types								
	HPV16, 66	2 (0.6)					1 (0.3)	3 (0.9)	
	HPV11, 16, 53, 81, 61	1 (0.3)						1 (0.3)	
	HPV positive	6 (1.9)	1 (0.3)	2 (0.6)	2 (0.6)	1 (0.3)	1 (0.3)	13 (4.1)	
	HPV negative	137 (43.2)	6 (1.8)					143 (45.1)	
	Total (HPV positive and negative)	143 (45.1)	7 (2.1)	2 (0.6)	2 (0.6)	1 (0.3)	1 (0.3)	156 (49.2)	

Positive	Single type								
	HPV11	2 (0.6)	1 (0.3)	2 (0.6)	1 (0.3)	1 (0.3)		7 (2.2)	0.001^∗^
	HPV16	1 (0.3)			1 (0.3)		1 (0.3)	3 (0.9)	
	HPV66				1 (0.3)			1 (0.3)	
	HPV81	21 (6.6)	5 (1.6)	12 (3.8)	6 (1.9)	4 (1.2)	3 (0.9)	51 (16.1)	
	Multiple types								
	HPV11, 66				1 (0.3)			1 (0.3)	
	HPV81, 44				1 (0.3)			1 (0.3)	
	HPV81, 88				1 (0.3)			1 (0.3)	
	HPV9, 53				1 (0.3)			1 (0.3)	
	HPV16, 58				1 (0.3)			1 (0.3)	
	HPV16, 66	3 (0.9)	1 (0.3)					4 (1.2)	
	HPV66, 53			1 (0.3)				1 (0.3)	
	HPV11, 45, 52, 87	1 (0.3)						1 (0.3)	
	HPV positive	28 (8.8)	7 (2.1)	15 (4.7)	14 (4.4)	5 (1.6)	4 (1.2)	73	
	HPV negative	68 (21.4)	20 (6.3)					88 (27.8)	
	Total (HIV positive and negative)	96 (30.3)	27 (8.5)	15 (4.7)	14 (4.4)	5 (1.6)	4 (1.2)	161 (50.8)	
Total (HIV positive and negative)		239 (75.4)	34 (10.7)	17 (5.4)	16 (5.0)	6 (1.8)	5 (1.5)	317 (100.0)	

ASCUS: atypical cells of unknown significance; CIN: cervical intraepithelial neoplasia; ICC: invasive cervical cancer; ^∗^: the probability at the 0.001 level.

## Data Availability

The datasets are available from the corresponding author on reasonable request.
